# Attribution and driving force of nitrogen losses from the Taihu Lake Basin by the InVEST and GeoDetector models

**DOI:** 10.1038/s41598-023-34184-x

**Published:** 2023-05-08

**Authors:** Xinghua He, Jiaming Tian, Yanqin Zhang, Zihan Zhao, Zucong Cai, Yanhua Wang

**Affiliations:** 1 School of Geography, Nanjing Normal University, 1 Wenyuan Road, Qixia, Nanjing, 210023 China; 2 Jiangsu Center for Collaborative Innovation in Geographical Information Resource Development and Application, Nanjing, 210023 China; 3 Key Laboratory of Virtual Geographic Environment, Ministry of Education, Nanjing Normal University, Nanjing, 210023 China

**Keywords:** Ecology, Environmental sciences

## Abstract

Quantifying temporal and spatial changes in reactive nitrogen (Nr) losses from a watershed and exploring its main drivers are the key to watershed water quality improvements. Huge Nr losses continue to threaten the safety of the water environment in the Taihu Lake Basin (TLB). Here, the InVEST and GeoDetector models were combined to estimate Nr losses in the TLB from 1990 to 2020 and explore driving forces. Different scenarios for Nr losses were compared, showing that Nr loss peaked at 181.66 × 10^3^ t in 2000. The key factors affecting Nr loss are land use, followed by elevation, soil, and slope factors, and their mean *q-*values were 0.82, 0.52, 0.51, and 0.48, respectively. The scenario analysis revealed that Nr losses increased under the business-as-usual and economic development scenarios, while ecological conservation, increased nutrient use efficiency, and reduced nutrient application all contribute to a reduction in Nr losses. The findings provide a scientific reference for Nr loss control and future planning in the TLB.

## Introduction

Excessive reactive nitrogen (Nr) loss is one of the greatest threats to aquatic ecosystems globally^1–3^. Anthropogenic activities, such as agricultural fertilization, industrial production, and sewage discharge, have accelerated the transport of terrestrial Nr to the aquatic ecosystems, i.e., rivers and lakes, resulting in eutrophication^4–6^, algal bloom^7^, greenhouse gas emission, and so on^3,8^. These phenomena severely threaten domestic water security and productive water supply. According to the world water development report, nearly six billion people worldwide will suffer from a clean water scarcity crisis by 2050^9^. In particular, water shortages are exacerbated by inadequate water quality in China, and a high Nr loading is one of the most severe threats to water quality^10^. There is an urgent need to reduce and control Nr losses.

In the past few decades, many studies on Nr loss focused on field measurements^11–16^. However, long-term watershed water quality monitoring and research are time-consuming and not suitable for assessing the impact of future land use change^14^. With the development of geographical information systems (GIS) and remote sensing, many models for assessing water quality and hydrological ecosystem services, such as the Soil and Water Assessment Tool (SWAT), Hydrological Simulation Program-FORTRAN (HSPF), Annualized Agricultural Non-Point Source (AnnAGNPS) and Integrated Valuation of Ecosystem Services and Trade-offs (InVEST) have been developed and are widely used^14–21^. Models based on nutrient transport processes such as SWAT, HSPF, and AnnAGNPS can simulate hydrological processes well and provide accurate results. However, these models require large amounts of hydrological data for calibration^18,22^, which is often not applicable in areas where data are scarce, and they are complex to operate and require specific training for operators^19,23^. The InVEST model contains fewer integrated algorithms than the other models and is, therefore, easier to use, allowing for modeling and analysis in areas where data are scarce, with low run times and strong spatial representation^24^. The nutrient delivery ratio (NDR) module in the InVEST model uses a more straightforward mass balance approach to describe the long-term and stable spatial movement of nutrients, which can not only reflect nutrient export under different climates and land use change scenarios but also help assess the impact of natural factors and human activities on pollution at the sub-basin or hydrological unit scale^25^.

Agricultural fertilization and urban wastewater discharge directly expose Nr to the environment^26^; climate and topography contribute to Nr loss by driving Nr transport^11^; changes in land use composition and structure can also affect Nr loss by altering surface nutrient content and nutrient transport^27^. Research has shown that the Nr loss has increased 3.5-fold due to agricultural intensification^28^, and increased impervious surfaces have also exacerbated Nr losses^29^. Moreover, the shift from forest to cleared land increases the denitrification capacity^28^; and increasing the patch edges of grassland can reduce Nr pollution^30^. However, traditional research tools and methods such as multiple linear regression, correlation analysis, ordinary least squares models, spatial lag models, and spatial error models are challenging to deal with drivers' spatial variability. They are limited to continuous data analysis, making it difficult for land use data to be used to analyze their direct effects on Nr loss. The GeoDetector model using continuous and typological data is an emerging statistical method that can reveal the drivers of spatial heterogeneity. It is based on the principle that if an independent variable significantly affects a dependent variable, then the spatial distribution of the independent and dependent variables should have similarity^31,32^. The GeoDetector model has been applied to various disciplines in nature and society^31^. For example, Chen et al. used the GeoDetector model to explore the driving role of factors such as land use on the spatial and temporal divergence of water resources in the Beijing-Tianjin-Hebei urban agglomeration^33^. Liu et al.^34^ explored the effects of nature, economy, and transportation on urban expansion based on the GeoDetector model.

Located in China’s most developed eastern region, the Taihu Lake Basin (TLB) covers only 0.4% of China's land but supports 4.4% of its population and contributes 9.7% of its GDP^35^. The high rates of economic development and intensive agricultural activities have accelerated the deterioration of water quality in the area^36–38^. As the lowest part of the basin, Taihu Lake receives a large amount of Nr. About 70,000 tonnes of riverine Nr entered Taihu Lake in 2009. Between 1998 and 2007, large amounts of cyanobacteria were present in the lake throughout the year, except in January and February^39^. A massive algal bloom led to a drinking water crisis in Wuxi in May 2007^40^, where almost half of Taihu Lake was covered by a cyanobacterial bloom, covering an area of over 1000 km^2^, resulting in over two million people being without drinking water for a week at the time^41^. In recent years, the water quality of Taihu Lake has improved due to the government's action and the implementation of the Overall Program on the Integrated Regulation of Taihu Lake; however, it is still moderately nutritious^42^. According to the reports, 39,500 tons of Nr were imported into Taihu Lake through rivers in 2018, and 20 of the 22 major rivers surrounding the lake had Nr concentrations above 2 mg L^−1^. Only 29.09% of the water quality sample sites assessed for potable water quality met the required standards^43^. Significant Nr losses still threaten the safety of the regional water environment. However, the current studies on Nr loss in Taihu Lake Basin are more limited to small scales such as fields^12,13^, and fewer studies have been conducted to investigate the spatial and temporal variation of Nr loss from the whole basin scale and to investigate the factors of Nr loss. Therefore, based on all the abovementioned issues, we first integrate the InVEST model’s NDR module with the GeoDetector model to explore the main drivers of Nr loss in the TLB. In addition, we designed seven scenarios to investigate the variation of Nr loss in TLB under different scenarios. The study aims to: (1) compare the Nr loss changes in the TLB during 1990–2020, (2) explore the hotspots of Nr loss and their spatial–temporal variations in the basin, (3) identify the main driving factors for the Nr cascade, and (4) predict future change characteristics of Nr losses in the basin under different scenarios.

## Materials and methods

### Description of the study area

**Figure 1:**
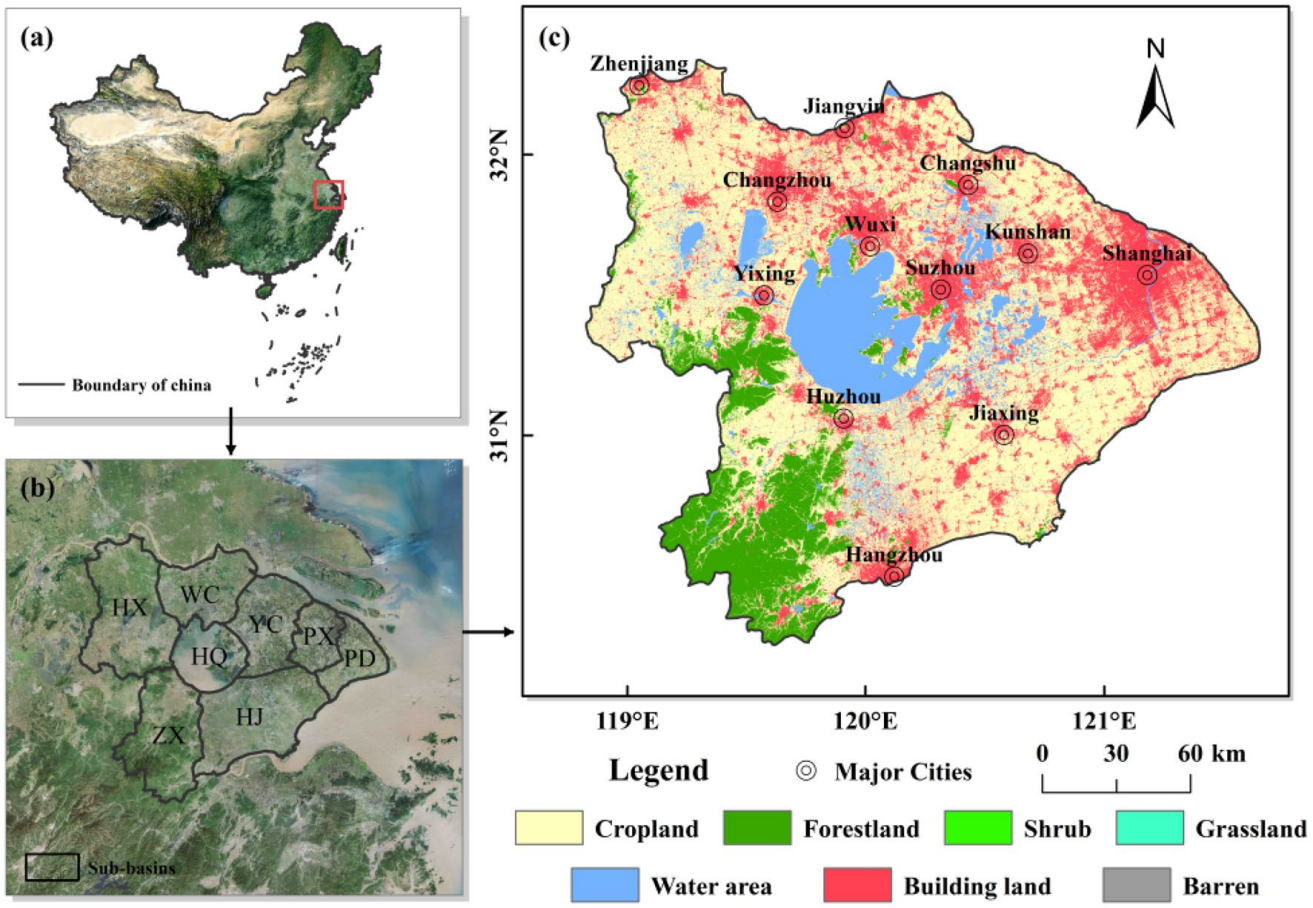
Location of the Taihu Lake Basin in China; (**a**) the specific location; (**b**) boundaries of the eight sub-basins of the basin; (**c**) land use type and location of major cities in the basin in 2020.

The TLB is located in eastern China (118°–121° E, 30°–33° N), within the core area of the Yangtze River Delta (Fig. 1). The basin covers portions of two provinces, namely Jiangsu and Zhejiang, as well as one prefecture-level city, Shanghai, encompasses several urban areas, including Suzhou, Wuxi, Changzhou, Zhangjiagang, and Zhenjiang. It is one of the most industrialized and urbanized regions in China, with a total area of about 36,900 km^2^. It has a humid northern subtropical climate zone with an average annual rainfall of 1177 mm and an average temperature of 16.2 °C. The basin has many rivers and a dense network of waterways, and it is well known as the land of fish and rice^6^. According to the topographic features and hydrological characteristics, the TLB can be divided into eight sub-basins (Fig. 1b), named Hu Xi (HX), Wu Cheng Xi Yu (WC), Yang Cheng Dian Liu (YC), Tai Hu (HQ), Zhe Xi (ZX), Hang Jia Hu (HJ), Pu Xi (PX), and Pu Dong (PD)^12^.

### The NDR module of the InVEST model

The NDR module in the InVEST model was used to simulate the Nr losses from the TLB, which formulas are shown in Eqs. (1, 2). Based on a DEM extraction of the river, the real river network was used to modify the original DEM data to further improve the accuracy of the model simulation (S1 and Fig. S1 in the Suppl. Mat.).

1 $$Load_{surf,i} = (1 - \Pr op_{subs,i} ) \times Load_{i}$$

2 $$Load_{surf,i} = \Pr op_{subs,i} \times Load_{i}$$

where $Load_{surf,i}$ and $Load_{subs,i}$ represent the surface and underground nutrient loads, respectively, $Load_{i}$ represents the Nr load of the grid unit, $\Pr op_{subs,i}$ refers to the ratio of underground nutrient runoff, and *i* is the grid unit.

The surface and underground nutrient transport rates were calculated by Eqs. (3, 4), respectively.

3 $$NDR_{surf,i} = NDR_{o,i} \left( {1 + \exp \left( {\frac{{IC_{i} - IC_{0} }}{k}} \right)} \right)^{ - 1}$$

4 $$NDR_{subs,i} = 1 - eff_{subs} \left( {1 - e^{{ - \frac{5l}{{l_{subs} }}}} } \right)$$

where $NDR_{surf,i}$ is the mobility rate of surface nutrients, $IC_{0}$ is the terrain index, $IC_{i}$ and *k* are the calibration parameter, $NDR_{o,i}$ is the proportion of unreserved nutrients in downstream pixels, $NDR_{subs,i}$ is the migration rate of underground nutrients, $eff_{subs}$ is the maximum nutrient retention efficiency that can be achieved through underground flow, $l_{subs}$ is the distance at which soil nutrient retention reaches its maximum capacity, and $l$ is the distance from the grid unit to the flow. Equations (5) and (6) were used to calculate the nutrient load.

5 $$x_{\exp ,i} = Load_{surf,i} \times NDR_{surf,i} + Load_{subs,i} \times NDR_{subs,i}$$

6 $$x_{{\exp_{tot} }} = \sum\limits_{i = 1}^{n} {x_{\exp ,i} }$$

where $x_{\exp ,i}$ represents the nutrient load of the grid cell *i*, and $x_{{\exp_{tot} }}$ refers to the total nutrient load in the basin.

In addition to the spatial data in Table 1, the NDR module also needs to provide data on the Nr loading for each land use type (S2 and Table S1 in the Suppl. Mat.), the retention efficiency of Nr for each land type, and the maximum Nr transport distance for each land type, as well as settings for the K parameter. Referring to the InVEST User's guide, the Nr load of the cropland in this study was derived from the Nr fertilization of the farmland in the region, which was obtained from the Statistical Yearbook.

**Table 1:** Data sources and processing.

Data	Source	Applied models
Precipitation (PRE)	National earth system science data center. http://www.geodata.cn/	NDR; GD; PLUS
Mean temperature		PLUS
Digital Elevation Model (DEM)	Geospatial Data Cloud. http://www.gscloud.cn	NDR; GD; PLUS
Soil types (SOIL)	Harmonized World Soil Database (version 1.1). https://iiasa.ac.at/models-and-data/harmonized-world-soil-database	GD
Boundary Taihu lake Basin	National cryosphere desert data center. http://www.ncdc.ac.cn/portal/	NDR
River network vector data	National Catalog Service For Geographic Information. https://www.webmap.cn/commres.do?method=result25	NDR
Land use/land cover (LUCC)	Zenodo Database, The 30 m annual land cover datasets and its dynamics in China from 1990 to 2021(Yang and Huang., 2021). https://zenodo.org/record/5816591#.Y3j44dhBxPZ	NDR; GD; PLUS
Slope (SLOPE)	Derived from the DEM data	GD; PLUS
Population (POP)	WorldPop. https://hub.worldpop.org/geodata/listing?id=29	GD; PLUS
Gross Domestic Product (GDP)	Figshare Database, Global 1 km × 1 km gridded revised real gross domestic product and electricity consumption during 1992–2019 based on calibrated nighttime light data. (Chen et al. 2022)	GD; PLUS
River network density (RND)	Derived from river network vector data, calculation reference (Liu et al. 2019)	GD; PLUS
Fractional Vegetation Cover (FVC)	Based on the Google Earth Engine platform, using a series of Landsat remote sensing images calculated based on the dichotomous model, the specific formula can be referred to (Zhang et al. 2022)	GD; PLUS
Proximity to highway	OpenStreetMap (https://www.openstreetmap.org/)	PLUS
Proximity to railway		
Proximity to highway		
Proximity to trunk roads		
Proximity to the primary road		
Proximity to the secondary road		
Proximity to the tertiary road		
Proximity to railway stations		

The Nr load from building land, including industrial and household, was obtained from the Overall Program on Integrated Regulation of the Taihu Lake Basin Water Environment^35^. The Nr load of other land use types and the removal efficiency of all land types were obtained from the InVEST user’s guide and details in Table 2^44^. The maximum Nr transport distance was set with a default value of 150 m. The InVEST model is sensitive to the K parameter^45^, which is the main parameter used for calibration. So we calibrated the model by adjusting the K parameter based on the observed Nr loss. Finally, the value of K was set to 12.5.

**Table 2:** Nr loading and removal efficiency for different LUCC types.

LUCC	Year	Load_n (kg N ha^−1^)	EFF_n (%)
Cropland	1990	185.31	30
	2000	216.43	
	2005	209.36	
	2010	174.20	
	2015	171.30	
	2020	158.78	
Forest	1990–2020	9.62	80
Shrub		2.12	70
Grassland		10	40
Water area		8.5	5
Barren		10	5
Developed		174.68	5

### Identification of the cold-hot spots in space

The Getis-Ord Gi* statistic is used to identify the low-value clusters (cold spots) and high-value clusters (hot spots) of regions^30,46,47^. In the present study, the Nr loss in each grid was extracted by creating a 1500 × 1500 m grid covering the study area, and the extracted data were analyzed for cold-hot spots.

### Determination of Nr loss drivers

The GeoDetector model system consists of four main components: Risk Detector, Factor Detector, Ecological Detector, and Interaction Detector. In this paper, we mainly use the Factor detector to explore the main driving forces of Nr loss.

The Factor Detector was applied to detect the degree of influence of the different factors on the Nr losses, measured by *q*-values with a range of [0,1]. The larger the *q*-value, the stronger the explanatory power. The formulas are as follows:

7 $$q = 1 - \frac{{\sum\nolimits_{h = 1}^{L} N_{h} \sigma_{h}^{2} }}{{N\sigma^{2} }} = 1 - \frac{SSW}{{SST}}$$

8 $$SSW = \sum\limits_{h = 1}^{L} {N_{h} \sigma_{h}^{2} } ,\,\,\,\,SST = N\sigma^{2}$$

where *h* = 1, … *L*, *L* is the classification of different factors; $N_{h}$ and $N$ refers to the number of units of layer *h* in each factor and the stratification of that factor, respectively; $\sigma_{h}^{2}$ and $\sigma^{2}$ represent the variance of layer *h* in each factor and the variance of the overall regional Nr losses, respectively, and SSW and SST are the sum of variances within layers and the overall total variance, respectively.

We synthesized previous studies^20,48,49^ and grouped the drivers into four categories of natural, socioeconomic, land use and composition, and landscape configuration, with 17 indicators chosen to explore the main drivers of the Nr losses (Table 3). We calculated all landscape composition and configuration indices using the ‘moving window’ function in Fragstats 4.2, which is a spatial statistics tool specifically designed to analyze and evaluate landscape patterns, and more detailed information can be obtained from its official website (https://fragstats.org/). All methods for optimal spatial discretization of the drivers and the number of discretizations are based on the ‘GD’ package in the R 4.2.2 software.

**Table 3:** Driving factors for Nr loss.

Category	Indicator	Abbreviations
Natural factors	Precipitation	PRE
	Elevation	DEM
	Slope	SLOPE
	Soil type	SOIL
	Fraction vegetation cover	FVC
	River network density	RND
Socioeconomic factors	Population density	PPO
	Gross domestic product	GDP
Land use and composition	Land use/land cover	LUCC
	Cropland proportion	CLP
	Forestland proportion	FLP
	Building land proportion	BLP
	Water area proportion	WAP
Landscape configuration	Contagion index^50^	CONTAG
	Shannon’s Diversity index^50^	SHDI
	Aggregation index^50^	AI
	Effective Mesh Size index^51^	MESH

### The PLUS model and scenario simulation

The Patch-generating Land Use Simulation (PLUS) model was applied to simulate future land use/land cover change (LUCC) in the TLB (S3 in the Suppl. Mat.). In the model protocol, the LUCC over two different periods was analyzed. Then, the LUCC expansion fraction for each land use type and the corresponding fraction for each driver was extracted and sampled. The random forest algorithm was used to quantify the influences and contribution of each driver to the expansion of each land use type and to obtain the development probability^52^. Finally, the model combined a cellular automata (CA) model with random seeds and a decreasing threshold mechanism to simulate the changes of each LUCC type with a high simulation accuracy^53–55^.

The probability of development for each LUCC type based on its change from 2015 to 2020 and 14 driving factors was calculated and assessed. The one in 2015 was chosen as the basis for simulating and predicting that in 2020. The observed LUCC in 2020 was used to verify the simulation results, and the kappa coefficient was selected to evaluate the accuracy of the simulation results. Generally, a kappa coefficient greater than 0.7 shows a high model accuracy^25,56^.

According to the development trend of the TLB, three land use scenarios of business-as-usual (BAU), ecological conservation (EC), and economic development (ED) were established in this study to simulate land use demand in 2035. The development probabilities of each LUCC type in the three land use scenarios were the same as in 2015–2020. The LUCC in 2020 is used as the basis for simulating and predicting 2035. The development probabilities of each LUCC type in the three land use scenarios are the same as in 2015–2020. Two constraint scenarios were added to explore the effects of reduced Nr fertilizer application (RNA) and increased Nr use efficiency (INUE) on the Nr losses. Thus, a combination of binding constraint scenarios and land use scenarios was considered. The scenarios were set up as follows.

1. BAU: This scenario maintained the historical trend based on a Markov chain projection of the BAU land demand in 2035.
2. ED: In this scenario, the development of the economy in the TLB takes priority. The reclamation of farmland and further urban expansion was encouraged. We calculate the land demand of 2035 in this scenario by modifying each category’s transfer matrix and transfer probabilities (Table S2). Specifically, the probability of transferring water area, barren grassland, and forestland to cropland was increased by 60%. The conversion probability of water area, barren, grassland, forestland, and cropland to urban land was increased by 100%.
3. EC: In this scenario, the future development of the TLB gives priority to ecological protection, with the government implementing the policies, i.e., forest protection, afforestation, and wetland restoration, while urban construction is prohibited from occurring on forestland and water bodies. We calculate the land demand of 2035 in this scenario by modifying each land use types transfer matrix and probability, including doubling the probability of converting cropland to both forestland and water bodies (Table S3). The conversion probability of barren to both forestland and water area increased by 80%. Meanwhile, we adjusted the probability of converting forestland and water area to building land to zero and reduced the probability of converting forestland and water area to other land use types by 40%.
4. RNA: This scenario followed the 2020 land use pattern. A reduction of 30% in Nr fertilizer application in 2020 was simulated.
5. INUA: This scenario followed the 2020 land use pattern. The retention rate of Nr nutrients in cropland increased from 30 to 40% in the simulation.
6. BAU + INUA: In this scenario, we combine the UBA and INUA scenarios to explore the impact of business-as-usual land use in 2035, with increased N use efficiency on N loss.
7. ED + INUA: In this scenario, we combine the ED and INUA scenarios to explore the impact of economic development land use in 2035, with increased N use efficiency on N loss.

### Data sources, statistical analysis, and visualization

The NDR module, PLUS model, and GeoDetector model required spatial grid data, i.e., a digital elevation model (DEM) and LUCC data. The data sources and preparation process are shown in Table 1.

Data processing and statistical analysis were conducted using Origin 2021 (OriginLab, Palo Alto, CA) and Excel 2019 (Microsoft Corp., Redmond, WA). The geodata spatial visibility and cold-spot calculations were completed with ArcGIS 10.08 (ESRI, Redlands, CA). The GeoDetector calculations were performed using the R version 4.2.2 (R Core Team, https://www.R-project.org/) ‘GD’ package^57^.

## Results

### Temporal and spatial variation of land use in the TLB during 1990–2020

**Figure 2:**
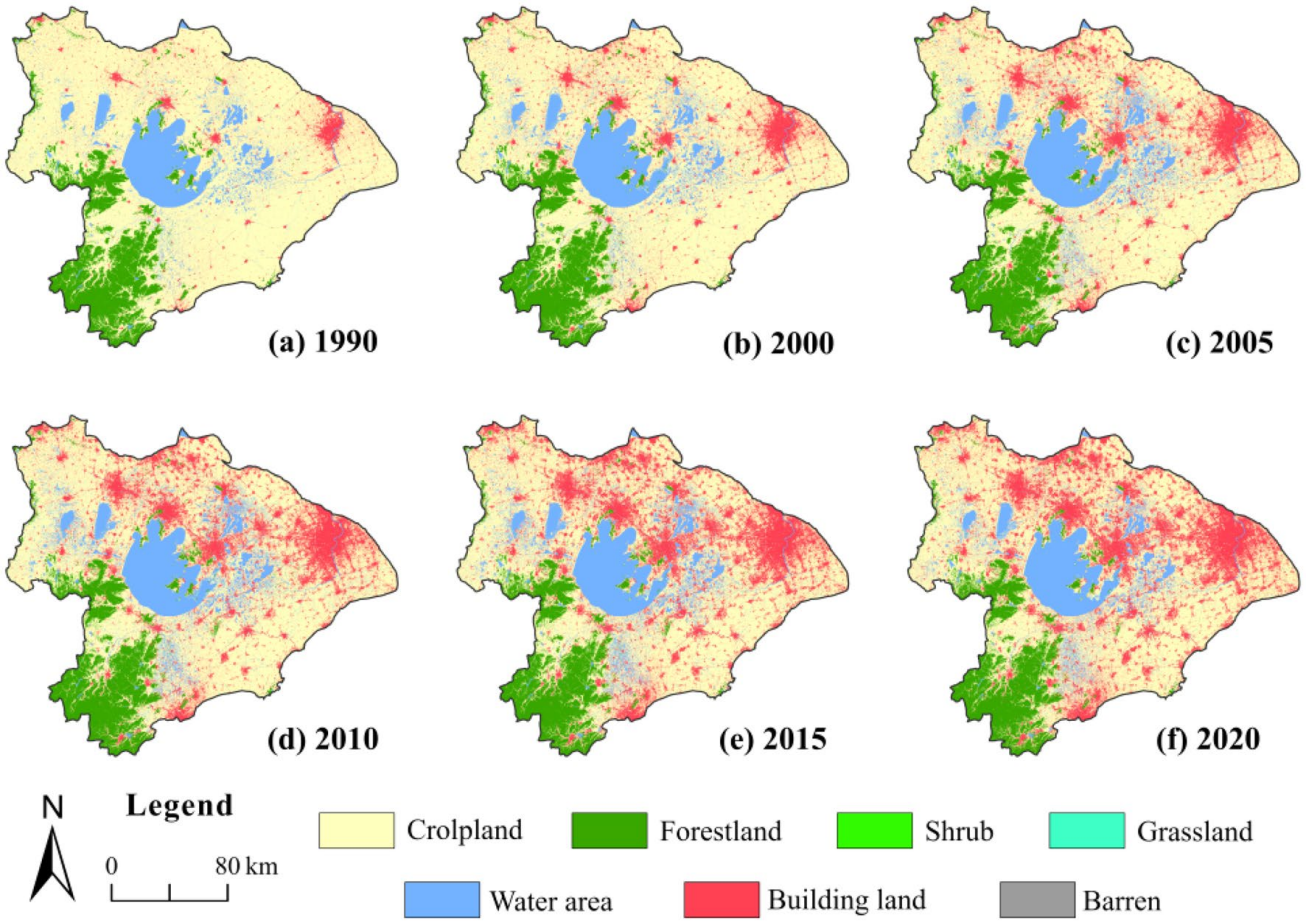
Changes in land use and land cover of the TLB from 1990 to 2020.

**Figure 3:**
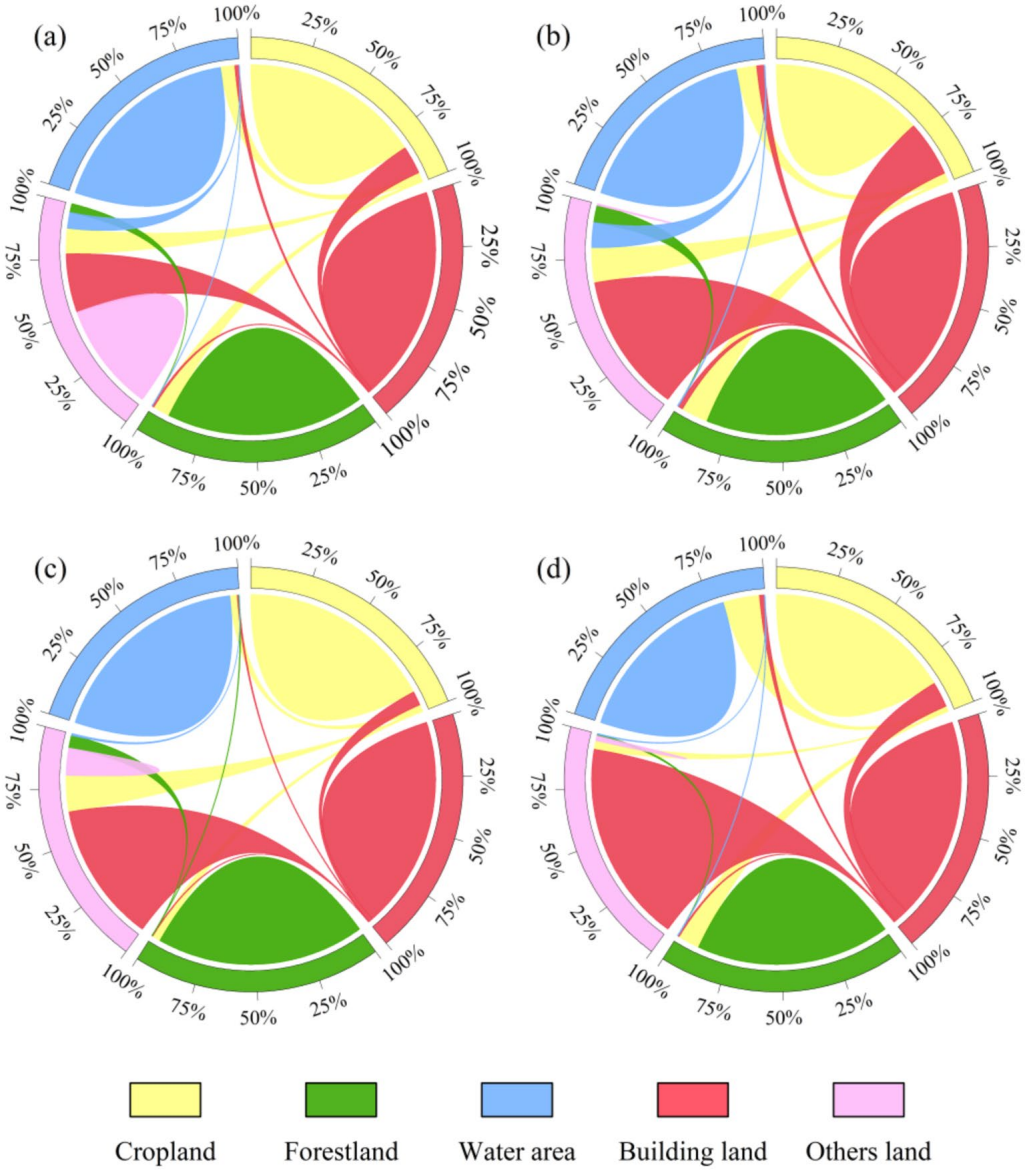
The transfer proportion chord diagram of land use and land cover for different periods of (**a**) 1990–2000, (**b**) 2000–2010, (**c**) 2010–2020, (**d**) 1990–2020. The width of the link between the different land classes represents the strength of the interchange between the two land classes during the period.

The land use structure of the TLB has changed dramatically in the past three decades, with a reduction of cropland and expansion of building land (Fig. 2). The transfer proportion chord diagram shows the conversion rate of cropland to building land showed an increase and then decreasing trend, which in the periods 1990–2000, 2000–2010, and 2010–2020 was 6.39%, 12.83%, and 11.57%, respectively (Fig. 3). And about 85% of the transferred cropland was converted into building land, resulting in a clear opposite trend in the area of both land use types (Fig. 3, Table S4). The cropland area decreased substantially from 70.53% in 1990 to 52.45% in 2020, but the building land area increased from 5.30% in 1990 to 23.71% in 2020 (Table 4).

**Table 4:** Temporal variation of the land use and Nr loss in the Taihu Lake Basin during 1990–2020.

Year	Nr loss (10^5^ t )	Cropland (%)	Forestland (%)	Water area (%)	Building land (%)	Other land use (%)
1990	1.59	70.53	12.87	11.29	5.30	0.02
2000	1.82	64.66	13.07	12.45	9.81	0.00
2005	1.75	59.88	12.45	13.50	14.14	0.02
2010	1.48	55.52	12.46	13.77	18.21	0.05
2015	1.50	53.06	11.73	13.42	21.78	0.01
2020	1.34	52.45	11.66	12.18	23.71	-

The forestland decreased slightly, mainly due to cropland reclamation, with approximately 18% of forestland converted to cropland from 1990 to 2020 (Fig. 3d). Although only 2.28% of forestland was directly converted to building land, the proportion of forestland converted to cropland also increased from 3.89% in 1990–2000 to 7.87% in 2000–2010 and 10.12% in 2010–2020 (Fig. 3). It was considered that accelerated urbanization indirectly led to a reduction in forestland (Fig. S2). The water bodies and other land use types also had the status of being transferred to each other.

Spatially, the land use changes were most apparent in the eastern and northern parts of the basin than in the south and west (Fig. 2). The expansion of building land and the reduction of cropland occurred mainly in the eastern and northern cities, i.e., Shanghai, Suzhou, Wuxi, Changzhou, and Zhangjiagang, which have developed into one of China’s major urban agglomerations over the past three decades (Figs. 1, 2). There was a decrease in forestland, mainly in the southwest, and the area of water bodies increased slightly in the south and west.

### Validation of the InVEST and PLUS models

According to the data published in the Overall Program on Integrated Regulation of Taihu lake basin Water Environment^35^, the amount of Nr entering the water bodies in the management area was 1.42 × 10^5^ t in 2005 and 1.35 × 10^5^ t in 2010. The simulation results of this study projected an Nr loss in the management area of 1.48 × 10^5^ t and 1.24 × 10^5^ t, respectively, comparable to the relative errors for the observed values of 5.65% and 7.02%. A report on the health of Taihu Lake provided by the Taihu Lake Basin Bureau found that the HX and HJ zones of the basin had a high level of Nr loss, which was consistent with our results (Fig. S3). The results of modeling the spatial distribution of Nr loss in this study were equally accurate.

The simulated 2020 land use was based on 2015 and validated its accurate reading using the observed 2020 land use (Fig. S4). The results show that the Overall accuracy is 0.89 and the Kappa coefficient is 0.84, indicating that the simulation results are highly accurate and can satisfy subsequent land use scenario simulations.

### Characteristics of the Nr loss associated with cold-hot spot identification in the TLB during 1990–2020

The Nr loss estimation results based on the InVEST model showed that from 1990 to 2000, the Nr losses in the TLB increased from 1.60 × 10^5^ to 1.82 × 10^5^ t. A decreasing trend was observed during 2000–2020, with a total of 1.34 × 10^5^ tons of Nr loss in 2020, indicating effective Nr control. Among the sub-basins, the HJ and HX zones had the highest Nr losses, accounting for 23–26% and 21–23% of the total losses each period, respectively (Fig. S5). The changes in Nr losses from each sub-basin were roughly the same as the overall Nr loss from the basin.

**Figure 4:**
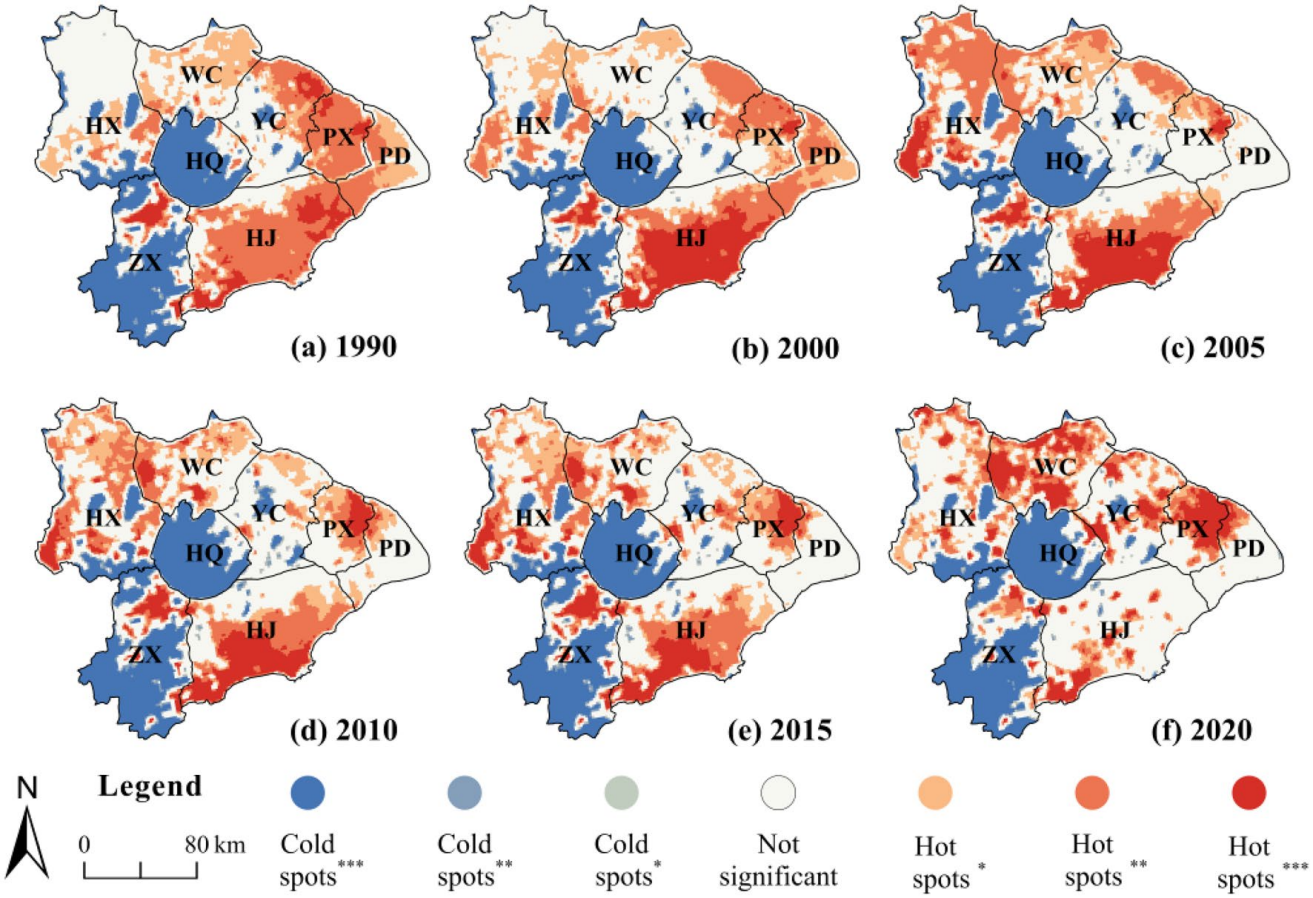
Distribution of the cold-hot spots for Nr loss from 1990 to 2020. *, **, and *** represent Nr loss at 90%, 95%, and 99% confidence intervals respectively; WC, HX, ZX, HQ, YC, PX, PD, and HJ represent eight sub-basins.

During the study period, the Nr loss cold spots were distributed in areas with clusters of water bodies and forestland. It was consistent with previous studies that found water bodies and forestland tended to be the regions with the lowest Nr loss^3^. However, the hot spots were more variable and gradually converged from the south and east to the north (Fig. 4). Most of the TLB area was a hot spot in 1990, except for the northern HX, western YC, and regions with clusters of water bodies and forestland (Fig. 4a). From 2000 to 2005, the hot spots in the north (HX and WC) gradually increased, while the hotspots in the east (YC, PX, and PD) and south (HJ) gradually decreased (Fig. 4b, c). During 2010–2020, hotspots in the south further reduced, while YC, WC, and PX displayed a noticeable clustering effect, with major cities such as Changshu, Suzhou, Wuxi, and Shanghai located in this area (Fig. 4d–f). Urban expansion may gradually become a significant contributor to Nr loss in the TLB.

### Attribution and driving forces of the Nr losses in the TLB

The explanatory power of potential drivers of Nr loss was calculated using GeoDetector to determine the main drivers and how these main drivers change over time (Table 5). The results show that all selected indicators passed the 5% significance test (*p* < 0.05) and we found that land use and composition were the primary drivers of the Nr losses in the TLB (Fig. S6, Table 5), which was consistent with previous research work^20,58–60^. The LUCC had the most substantial explanatory power for Nr loss, with* q*-values above 0.8 and a mean value of 0.82 over the study period (Table 5). Among its main components (cropland, forestland, water area, and building land), the *q*-values of the percentages of forestland (FLP) and water bodies (WAP) varied slightly. The* q*-values decreased from 0.38–0.29 in the FLP and increased from 0.30 to 0.32 in the WAP during 1990–2020. However, it is interesting to note that the *q*-values of cropland (CLP) percentages and building land (BLP) changed considerably and displayed opposite trends, which is consistent with the trend of cropland and urban land use change. The *q*-values of CLP decreased from 0.69 to 0.25 and BLP increased from 0.18 to 0.54.

**Table 5:** The* q*-value of different driving factors on Nr loss.

Driving factor	1990	2000	2005	2010	2015	2020
LUCC	0.84	0.84	0.83	0.81	0.80	0.81
DEM	0.57	0.55	0.52	0.49	0.49	0.48
SOIL	0.56	0.55	0.51	0.48	0.47	0.48
SLOPE	0.55	0.51	0.48	0.44	0.45	0.43
CLP	0.69	0.60	0.52	0.38	0.33	0.25
FLP	0.38	0.36	0.33	0.31	0.31	0.29
WAP	0.30	0.31	0.32	0.31	0.32	0.30
BLP	0.18	0.21	0.24	0.29	0.33	0.54
GDP	0.32	0.29	0.26	0.26	0.27	0.33
POP	–	0.27	0.27	0.28	0.26	0.34
FVC	0.30	0.20	0.19	0.23	0.20	0.25
MESH	0.14	0.16	0.17	0.17	0.19	0.23
CONTAG	0.14	0.16	0.17	0.17	0.19	0.26
AI	0.12	0.14	0.16	0.17	0.18	0.26
SHDI	0.10	0.13	0.15	0.15	0.17	0.23
RND	0.16	0.13	0.11	0.11	0.11	0.12
PRE	0.15	0.09	0.07	0.10	0.12	0.14

In the natural factors, the *q*-values for DEM, soil type (SOIL), and SLOPE showed a decreasing trend in their effects on the Nr losses over the period. But, they remained in the range of 0.4–0.6. The mean *q*-values for DEM, SOIL, and SLOPE were 0.52, 0.51, and 0.48, second only to LUCC, suggesting that the soil and geological environment still had a crucial influence on the Nr losses in the area. However, other natural factors such as precipitation (PRE), river network density (RND), and fraction vegetation cover (FVC) have lower *q*-values (< 0.3) and remained stable or decreased slightly (Table 5), indicating that precipitation, river network density, and vegetation cover do not significantly affect Nr loss. Among the socioeconomic factors, the *q*-values of gross domestic product (GDP) changed stably, and the population (POP) factor increased slightly. It is worth noting that although the indices characterizing land use structure, such as MESH, CONTAG, AI, and SHDI, had small *q*-values throughout the study period, they tended to increase, contrary to the tendency of most drivers to have progressively lower *q*-values. The effects of land use structure on the Nr losses in the TLB were gradually enhanced as the land use changes became more pronounced during the study period (Fig. 2, Table 4, Table 5).

### Scenario simulation and prediction of the Nr losses in the TLB

**Figure 5:**
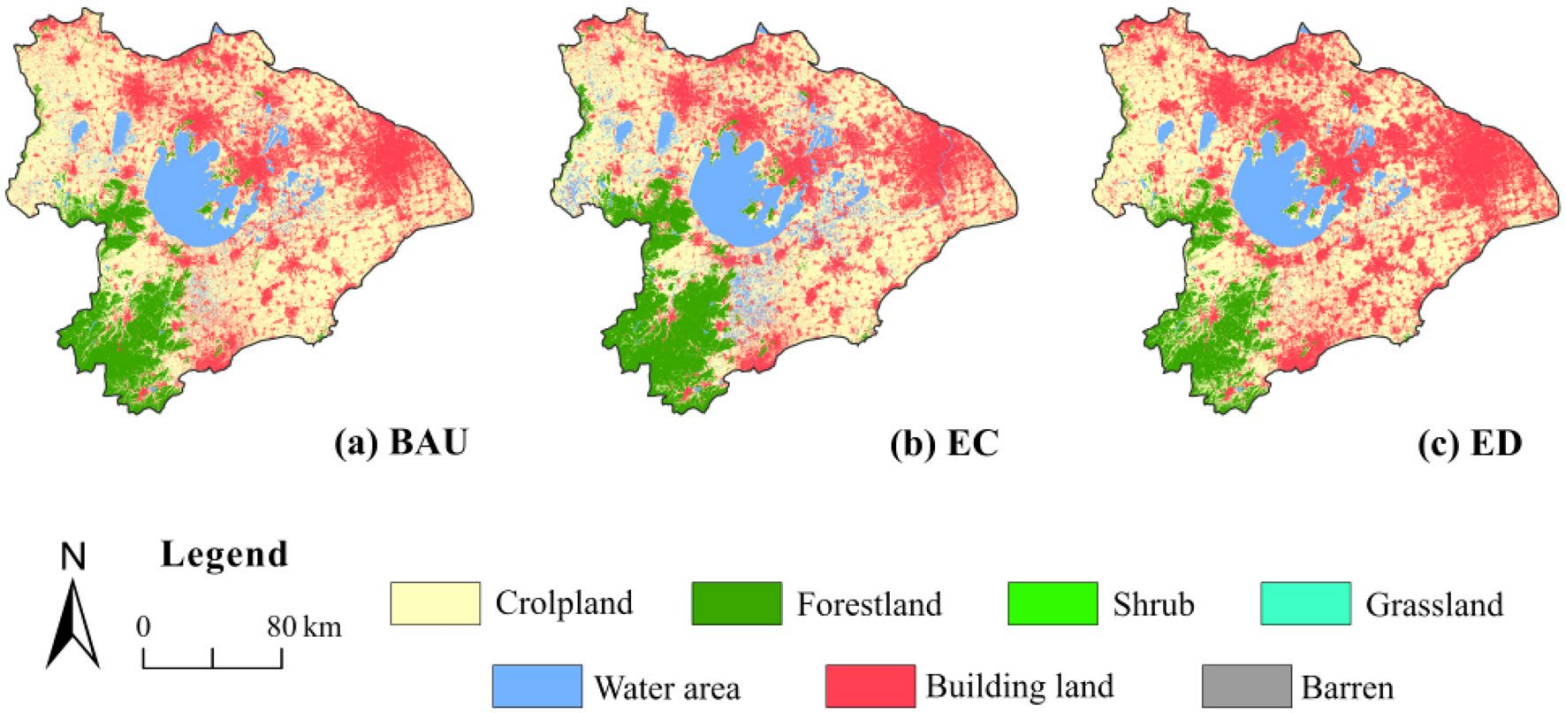
The land use and land cover distribution for different scenarios; BAU, business-as-usual; EC, ecological conservation; ED, economic development.

There were differences in the land use changes by 2035 among the three different land use scenarios (Fig. 5). In the BAU scenario, the proportion of cropland, forestland, and water bodies decreased, while the building land increased to 28.24%. In the EC scenario, the area of forestland and water increased (Table 6). In the ED scenario, the proportion of cropland, forestland, and water bodies decreased to 45.46%, 10.12%, and 7.12%, respectively, and the building land increased to 37.30%.

**Table 6:** Nr loss and land use percentage under different scenarios.

Scenario	Nr loss (10^5^t)	Cropland (%)	Forestland (%)	Water area (%)	Building land (%)
BAU	1.41	50.86	11.41	9.49	28.24
ED	1.52	45.46	10.12	7.12	37.30
EC	1.32	46.35	13.79	12.66	27.20
IUNA	1.17	52.45	11.66	12.18	23.71
RNA	1.09	52.45	11.66	12.18	23.71
ED+IUNA	1.36	45.46	10.12	7.12	37.30
BAU+IUNA	1.25	50.86	11.41	9.49	28.24

Spatially, in the BAU and ED scenarios, building land expands through the occupation of cropland, resulting in the more significant urban agglomeration in the north and west. The reduction of forestland occurred mainly in the southwest, and most of them converted to cropland. The decrease in water area occurs primarily in the western and southwestern regions. On the contrary, in the EC scenario, the increase in forestland is mainly in the southwest, while the increase in water area is mainly in the southwestern and west (Figs. 2f, 5b). Due to restrictions on the occupation of forestland and water area, the expansion of building land in this scenario mainly takes place on cropland (Table 6).

**Figure 6:**
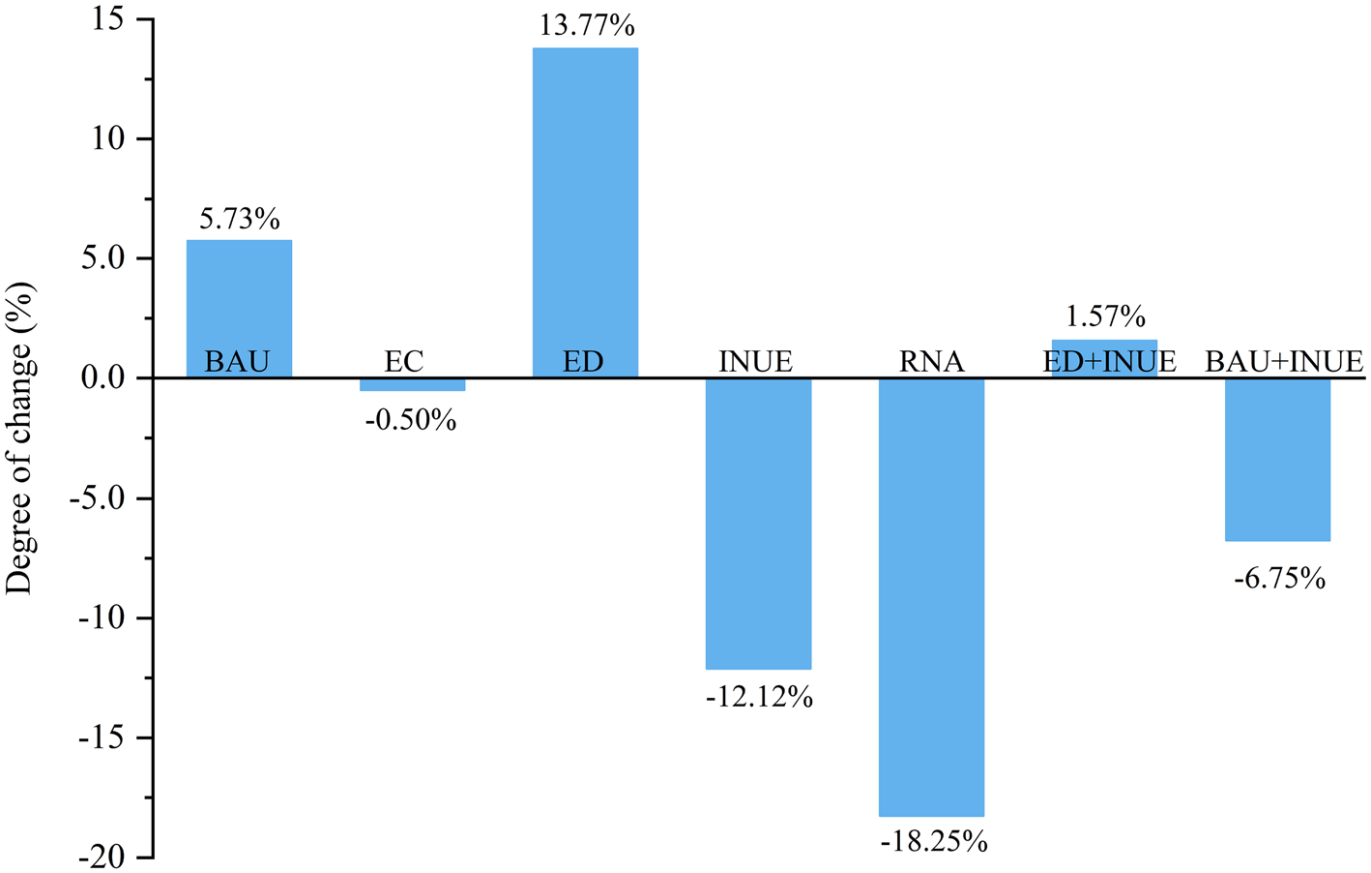
Nr loss changes under different scenarios. All scenario Nr loss changes are compared to 2020; BAU, business-as-usual; EC, ecological conservation; ED, economic development; INUE, increased Nr use efficiency; RNA, reduced Nr fertilizer application; ED + INUA, combining the ED and INUA scenarios; BAU + INUE, combine the UBA and INUA scenarios.

The Nr losses under the different scenarios were evaluated using the InVEST model. The spatial data, such as the DEM, precipitation, and the model parameters used in the simulation of the different scenarios, were the same as in 2020, which enabled the effects of the scenarios to be highlighted and a comparison of the results with those of 2020. A comparison of the Nr losses in 2020 among the different scenarios (Fig. 6) showed that the Nr losses in the BAU and ED scenarios increased by 5.73% and 13.77%, with the totals reaching 1.41 × 10^5^ t and 1.52 × 10^5^ t, respectively (Table 6). The Nr losses in three scenarios, i.e., RNA, INUA, and EC, decreased by 18.25%, 12.12%, and 0.50%, respectively. The combination of several scenarios, i.e., BAU, ED, and INUA, was more conducive to economic and technological improvements than the single land use change scenario and the constraints scenario. The Nr loss was reduced by 6.75% in the BAU-combined INUE scenario and increased by 1.57% in the ED-combined INUE scenario, showing that both BAU + INUA and ED + INUE scenarios significantly reduce Nr loss (Fig. 6)

## Discussion

### Effects of precipitation and topography on Nr loss in the TLB

Previous studies indicated that wet precipitation increased Nr loss through leaching and runoff mechanisms, thus was considered to be the primary factor driving Nr loss in farmland^11^. However, the *q*-value of rainfall observed in this study was relatively low, which can be attributed to the slight variation of rainfall in this region. Contrary to conventional belief, topography was believed to have less impact on Nr loss in flat areas because of fewer differences in terrainn^3^. However, our study demonstrates that topography, as described by the DEM and slope, exerts the second-highest degree of influence on Nr loss in the TLB, following LUCC (Table 5). This could be linked to farming practices at higher elevations and steeper slopes, where heavy applications of Nr fertilizer exacerbated Nr loss under the influence of topography (Figs. 2, S2a,b). Our hotspot analysis and statistical results also show that the ZX and HX regions, which have significant variations in topography, contribute a substantial amount of Nr export and persistent Nr loss hotspots (Figs. 4, S5). Therefore, to mitigate the impact of high topographic relief on Nr loss, it is essential to implement land management strategies that effectively transfer and manage farmland in these areas.

### Land use planning recommendations

Our research demonstrates the crucial role of land use and composition in Nr loss. Despite a decrease in Nr loss in the TLB over the last three decades, potentially resulting from the implementation of the Overall Program for the Comprehensive Management of the Water Environment in the TLB and decreased Nr fertilizer usage, current levels of Nr loss in the basin are still elevated, with an unsuitable land use structure dominated by croplands and urban areas. The Yangtze River Delta Urban Agglomeration Development Plan anticipates ongoing urban expansion across the basin. Urban development and intensive agricultural activities elevate nutrient outflow, rendering croplands and urban areas essential contributors to Nr losses^27^. Expanding building areas will generate more impervious surfaces, increasing urban runoff and heightening the risk of Nr loss^60–62^. Our simulation of land use scenarios in BUA and ED also reveals that urban development leads to deforestation and increased Nr loss. Several studies have shown that poor water quality is a major cause of rural poverty in China^63^, and that high levels of Nr loss add to the costs of environmental management with additional investments anticipated. Thus, developing appropriate land use strategies and management measures to balance economic growth with environmental preservation is imperative. Forestland and wetlands can reduce Nr export through sediment and nutrient retention^64,65^, as supported by our EC findings, which demonstrate that most of the restoration of these areas was achieved by converting croplands.

Our recommendations for farmland, forestland, and water planning based on our earlier analyses are as follows: (1) strengthening protective policies for steep-slope farmland, such as constructing terraced fields to minimize Nr loss from soil; (2) providing agricultural subsidies to incentivize farmers to reduce fertilizer usage or restore forestlands on steep-slope farmland; (3) successful implementation of riparian buffer protection measures in other instances suggests their potential effectiveness in reducing Nr loss^66^. The TLB features numerous rivers, making riparian buffer protection measures such as increasing the proportion of vegetation within buffer zones and incorporating ponds that consume Nr during migration, effectively reducing Nr loss. For urban, this study shows that the *q*-value of BLP rose to 0.54 and that of POP rose to 0.34 in 2020, which indicates that urban expansion and population growth have an increasing impact on Nr loss within the region, and urban areas are gradually becoming hotspots for Nr loss. Our recent research using Nr and oxygen isotopes to track the nitrate of water bodies in the TLB river network indicates that manure and sewage contribute the most to water body nitrate content^37^. Several studies of rivers or cities within the TLB also have shown that domestic and industrial wastewater made substantial contributions as Nr sources^37,48,67^. However, despite the TLB pioneering the advancement of wastewater treatment in China, its progress is being outpaced by urbanization and ongoing socioeconomic expansion. Its current wastewater treatment standards are inadequate to satisfy the environmental capacity of the watershed^68^. Hence, urban wastewater treatment standards in the region must be upgraded; at the same time, size and population need to be effectively controlled to accommodate the relatively poor wastewater recovery rates and discharge standards.

### Limitations and prospects

There are still some limitations in the methods and data used. First, due to issues with data availability, when using the InVEST model to simulate Nr loss over the past 30 years, the Nr load of croplands varied with different years, while that for other land covers was fixed. Although this approach is widely used in the model and we obtained reliable simulation results^22,45,68^, it may introduce some bias and uncertainty. Therefore, future studies should improve the Nr load for different periods to simulate Nr loss more accurately. Secondly, when predicting future scenarios of Nr loss, to better explore the impact of land use on Nr loss, we only changed land use under different scenarios in the model settings without considering the influence of meteorological factors on Nr loss. However, Nr loss is affected by various factors, and the actual situation may be more complex. Future studies should improve the settings for these factors. Finally, this study simulated and predicted Nr loss and its driving factors at the watershed scale. However, Nr loss varies and is complex at different scales. Therefore, in the future, we will consider analyzing and explaining Nr loss and attribution from multiple scales such as sub-watersheds, counties, altitude gradients, etc.

## Conclusions

In this study, we combined InVEST and GeoDetector models to simulate and study the Nr loss and influencing factors in the TLB and simulated seven possible scenarios. Changes in the Nr loss hot spots in the TLB and driving forces over the past 30 years were clarified and subsequently, the future Nr losses under various scenarios were predicted. The results show that from 1990 to 2020, the highest Nr loss from the TLB was found to be 1.82 × 105 t N in 2000, and the city gradually became a hot spot for Nr loss. Implementing ecological protection, increasing nutrient use, and reducing fertilizer application can reduce N loss. Land use percentage (BLP) on Nr loss increases gradually, reaching a *q-*value of 0.54 by 2020. Nr loss is also constrained by topography and soil, with average *q-*values of 0.52, 0.51, and 0.48 for elevation, soil, and slope. The influence of population factors on N loss is also increasing yearly. Based on the above conclusions, we suggest that watershed managers should strengthen the implementation of soil and water conservation measures on cropland in high and steep slope areas, implement an encouraging policy of returning farmland to forest and reduce Nr loss by establishing river buffer zones, while the size of cities and population needs to be controlled and the level of wastewater treatment needs to be improved. Our study can provide a scientific reference for watershed management. Further studies will be conducted for different sub-basins to provide more precise solutions for water pollution management.

## Supplementary Information

Supplementary Information.

## Data Availability

The datasets used and/or analysed during the current study available from the corresponding author on reasonable request.
