# Supplementary material for: Attribution and driving force of nitrogen losses from the Taihu Lake Basin by the InVEST and GeoDetector models
Source: Sci Rep. 2023 May 8;13:7440. doi: 10.1038/s41598-023-34184-x (PMC10167248; doi:10.1038/s41598-023-34184-x)
Supplement: Supplementary file 1 — Supplementary Information. [file 41598_2023_34184_MOESM1_ESM.docx]

**Supplementary Materials**

**Attribution and driving force of nitrogen losses from the Taihu Lake Basin by the InVEST and** **GeoDetector models**

Xinghua He^a^, Jiaming Tian^a^, Yanqin Zhang^a^, Zihan Zhao^a^, Zucong Cai^a,b,c^, Yanhua Wang^a,b,c^[[1]](#footnote-1)^*^

^a^ *School of Geography, Nanjing Normal University, Nanjing 210023, China*

^b^ *Jiangsu Center for Collaborative Innovation in Geographical Information Resource Development and Application, Nanjing 210023, China*

^c^*Key Laboratory of Virtual Geographic Environment, Ministry of Education, Nanjing Normal University, Nanjing 210023, China*

**Page S2−S16**

**Contents of this file**

**Section S1: Digital Elevation Model data correction.**

**Section S2: Land use /land cover data description.**

**Section S3**: **PLUS model.**

**Table S1.** Land use/land cover classes used in the maps for the Taihu Lake Basin.

**Table S2.** Business-as-usual (BAU) and economic development (ED) Scenarios land use transfer matrix and neighborhood weights.

**Table S3.** Ecological conservation (EC) Scenarios land use transfer matrix and neighborhood weights.

**Table S4.** Land Use Transfer Matrix for Taihu Lake Basin 1990-2000, 2000-2010, 2010-2020, 1990-2020

**Fig. S1** Comparison of original DEM and corrected DEM of TLB; (a) Original DEM; (b) Real river network; (c) Modified DEM.

**Fig. S2** Spatial distribution of driving factors affecting land use.

**Fig. S3** Mean nitrogen loss from sub-basins; WC, HX, ZX, HQ, YC, PX, PD, HJ represent eight sub-basins.

**Fig. S4** Comparison of observed land use and simulated land use.

**Fig. S5** Percentage of nitrogen loss from sub-basins; WC, HX, ZX, HQ, YC, PX, PD, HJ represent eight sub-basins.

**Fig. S6** Percentage of nitrogen loss from different land use types.

**Section S1**: **Digital Elevation Model data correction.**

The NDR in the InVEST model assumes that nitrogen loss stops transport when it reaches the river (Sharp et al., 2016). The model is based on a DEM extraction of the river, so DEM data that accurately reflects surface relief is important for accurate model simulation. Here, we use real river network data to modify the original DEM data to further improve the accuracy of the model simulations (Wang et al., 2022).

**Section S2**: **Land use /land cover data description.**

***Data sources and reliability.*** The land use (1990-2020) data for the Taihu lake basin (TLB) used in this study were derived from the China Land Use/Cover Dataset (CLCD) produced by (Yang and Huang., 2021). The data is based on 335,709 Landsat satellite images acquired by Google Earth Engine (GEE), combined with the Chinese land use/cover datasets (CLUDs) and visual interpretation to collect and train classification samples, and finally, a long time series continuous (1990-2020) Chinese land use land cover data was constructed using a random forest classifier. A total of nine land use types were included in the CLCD, including cropland, forest, shrub, grassland, water, snow, and ice, barren, impervious, and wetland, with a verified overall accuracy of 79.31%, outperforming MCD12Q1, ESACCI_LC, FROM_GLC, and GlobeLand30, with high reliability.

***Data processing.*** We obtained CLCD raster data for 1990, 2000, 2005, 2010, 2015, and 2020 from the Zenoda database (Yang and Huang., 2021); we used ArcGIS10.08 Clip Raster Tool to obtain the land use raster data of TLB for the corresponding years. There are seven land uses in the TLB part of CLCD, and we renamed them without changing their original data, as detailed in Table S1.

**Section S3**: **PLUS model.**

***Drivers of land use change.*** Land use change is constrained by a variety of natural and socio-economic factors, based on this we have selected 14 drivers for modeling land use change concerning previous studies.

***Neighborhood weights and transfer matrix.*** Within a unique neighborhood weight, different land use types may have different neighborhood effects. According to Liang et al., 2021, we calculated the proportion of the total area expanded by land use type from 2015-2020 and used this to determine the neighborhood weights for each land use type. The conversion matrix specifies whether land uses can be converted to each other, and we set different conversion matrices based on the needs of the scenario projections.

***Conversion*** ***constraint.*** The conversion constraint zone specifies whether land use changes are allowed within the rezoning area. In this study, we specified that large lake water bodies within the TLB remain unchanged and are not allowed to be occupied.

**Table S1.** Land use/land cover classes used in the maps for the Taihu Lake Basin.

| Names used in this study | Name in the CLCD | Description |
| --- | --- | --- |
| Cropland | Cropland | Cropland, rainfed; Cropland, irrigated or post-flooding; Mosaic cropland (>50%) / natural vegetation (tree, shrub, herbaceous cover) (<50%); Mosaic natural vegetation (tree, shrub, herbaceous cover)(>50%)/cropland. |
| Forestland | Forest | Tree cover, needle-leaved, evergreen, closed to open (>15%); Tree cover, needle-leaved, deciduous, closed to open (>15%); Tree cover, mixed leaf type (broadleaved and needle-leaved); Tree cover, broadleaved, evergreen, closed to open (>15%); Tree cover, broadleaved, deciduous, closed to open (>15%); Mosaic tree and shrub (>50%) / herbaceous cover (<50%); Mosaic herbaceous cover (>50%) / tree and shrub (<50%). |
| Shrub | Shrub | Shrubland. |
| Grassland | Grassland | Grassland; Sparse vegetation (tree, shrub, herbaceous cover)  (<15%); Lichens and mosses |
| Water area | Water | Water bodies |
| Barren | Barren | Bare areas |
| Building land | Impervious | Urban and built-up lands; Urban areas |

**Table S2.** Business-as-usual (BAU) and economic development (ED) Scenarios land use transfer matrix and neighborhood weights.

|  | Cropland | Forestland | Shrub | Grassland | Water area | Barren | Building land |
| --- | --- | --- | --- | --- | --- | --- | --- |
| Cropland | 1 | 1 | 1 | 1 | 1 | 1 | 1 |
| Forestland | 1 | 1 | 1 | 1 | 1 | 1 | 1 |
| Shrub | 1 | 1 | 1 | 1 | 1 | 1 | 1 |
| Grassland | 1 | 1 | 1 | 1 | 1 | 1 | 1 |
| Water area | 1 | 1 | 1 | 1 | 1 | 1 | 1 |
| Barren | 1 | 1 | 1 | 1 | 1 | 1 | 1 |
| Building land | 1 | 1 | 1 | 1 | 1 | 1 | 1 |
| Neighborhood weight | 0.045 | 0.068 | 0.0001 | 0.0001 | 0.008 | 0.0001 | 0.0406 |

Note: 1 and 0 represent interconvertible and not interconvertible, respectively.

**Table S3.** Ecological conservation (EC) Scenarios land use transfer matrix and neighborhood weights.

|  | Cropland | Forestland | Shrub | Grassland | Water area | Barren | Building land |
| --- | --- | --- | --- | --- | --- | --- | --- |
| Cropland | 1 | 1 | 1 | 1 | 1 | 1 | 1 |
| Forestland | 1 | 1 | 1 | 1 | 1 | 1 | 1 |
| Shrub | 1 | 1 | 1 | 1 | 1 | 1 | 1 |
| Grassland | 1 | 1 | 1 | 1 | 1 | 1 | 1 |
| Water area | 1 | 1 | 1 | 1 | 1 | 1 | 1 |
| Barren | 1 | 1 | 1 | 1 | 1 | 1 | 1 |
| Building land | 1 | 1 | 1 | 1 | 1 | 1 | 1 |
| Neighborhood weight | 0.045 | 0.068 | 0.0001 | 0.0001 | 0.008 | 0.0001 | 0.0406 |

Note: 1 represents interconvertible; 0 represents not interconvertible.

**Table S4.** Land Use Transfer Matrix for Taihu Lake Basin 1990-2000, 2000-2010, 2010-2020, 1990-2020

| Time period | Land use (ha) | Cropland | Forest | Impervious | Water | Others land |
| --- | --- | --- | --- | --- | --- | --- |
| 1990-2000 | Cropland | 2338410.70 | 165620.50 | 26498.96 | 40.35 | 58940.21 |
|  | Forest | 18326.61 | 1589.17 | 450185.32 | 16.99 | 430.10 |
|  | Impervious | 3386.36 | 182547.69 | 47.16 | 0.93 | 569.29 |
|  | Water | 14065.73 | 2667.12 | 1160.94 | 0.53 | 392638.99 |
|  | Others land | 87.58 | 334.65 | 31.10 | 64.43 | 4.20 |
| 2000-2010 | Cropland | 1963462.89 | 304683.87 | 19897.43 | 1513.78 | 84706.36 |
|  | Forest | 4574.84 | 345587.50 | 51.02 | 6.06 | 2554.32 |
|  | Impervious | 37620.25 | 4215.64 | 435383.27 | 27.98 | 676.15 |
|  | Water | 22.34 | 51.32 | 7.70 | 95.04 | 13.68 |
|  | Others land | 30412.37 | 8640.99 | 284.10 | 55.15 | 413184.51 |
| 2010-2020 | Cropland | 1742532.57 | 235080.09 | 19216.98 | 37.08 | 33749.91 |
|  | Forest | 44815.77 | 616313.43 | 410.31 | 11.25 | 4574.70 |
|  | Impervious | 46120.59 | 2849.40 | 405773.01 | 11.88 | 1018.71 |
|  | Water | 70.65 | 1616.22 | 13.14 | 38.25 | 5.85 |
|  | Others land | 84821.31 | 11254.50 | 905.13 | 22.77 | 406148.67 |
| 1990-2020 | Cropland | 1808039.52 | 662235.39 | 25631.55 | 67.59 | 83691.09 |
|  | Forest | 12097.53 | 179570.52 | 117.09 | 0.90 | 2152.71 |
|  | Impervious | 58720.86 | 10713.60 | 399445.83 | 39.87 | 1671.66 |
|  | Water | 90.90 | 359.37 | 45.00 | 3.87 | 63.99 |
|  | Others land | 39412.08 | 14234.76 | 1079.10 | 9.00 | 357918.39 |


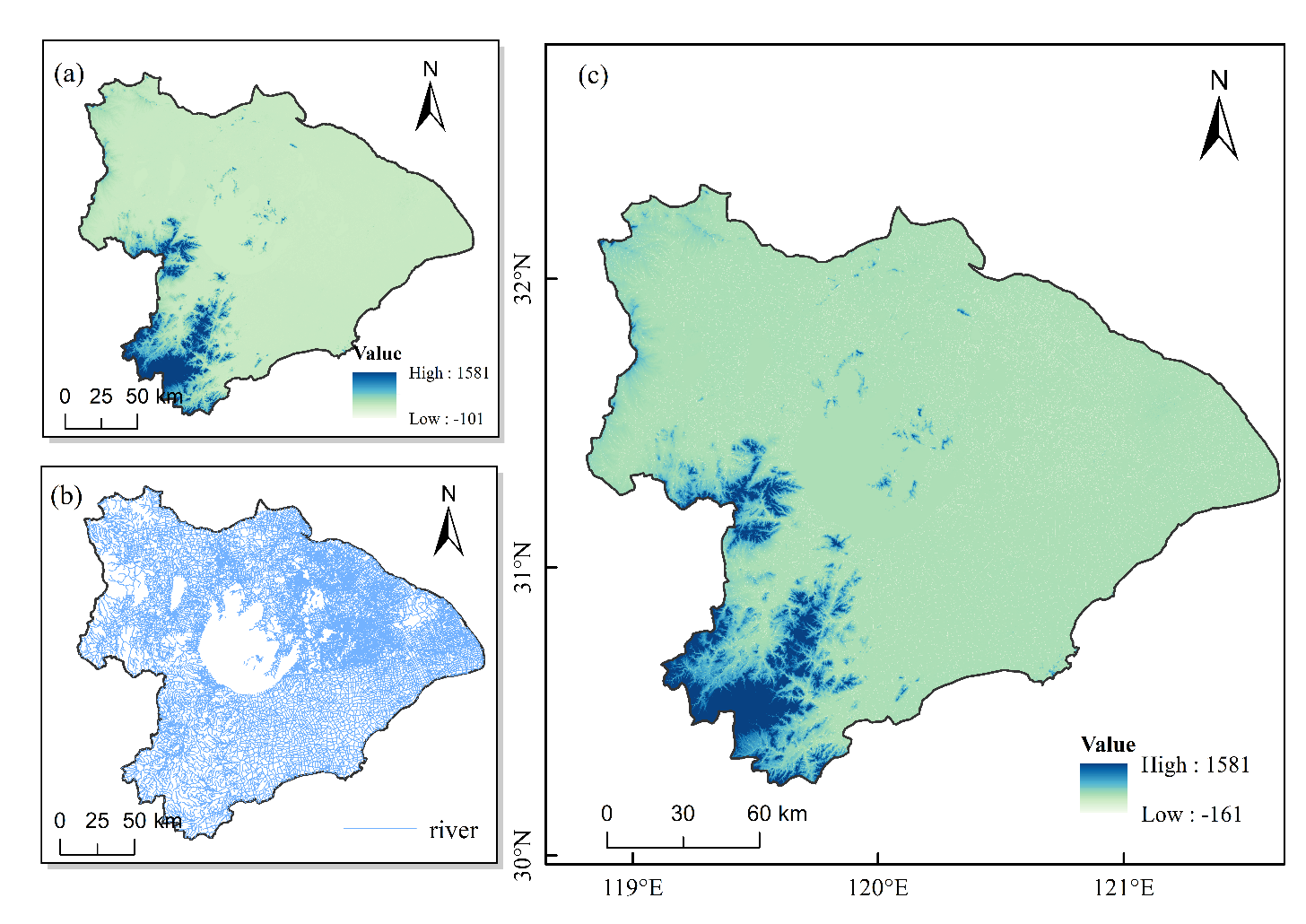


**Fig. S1** Comparison of original DEM and corrected DEM of TLB; (a) Original DEM; (b) Real river network; (c) Modified DEM.


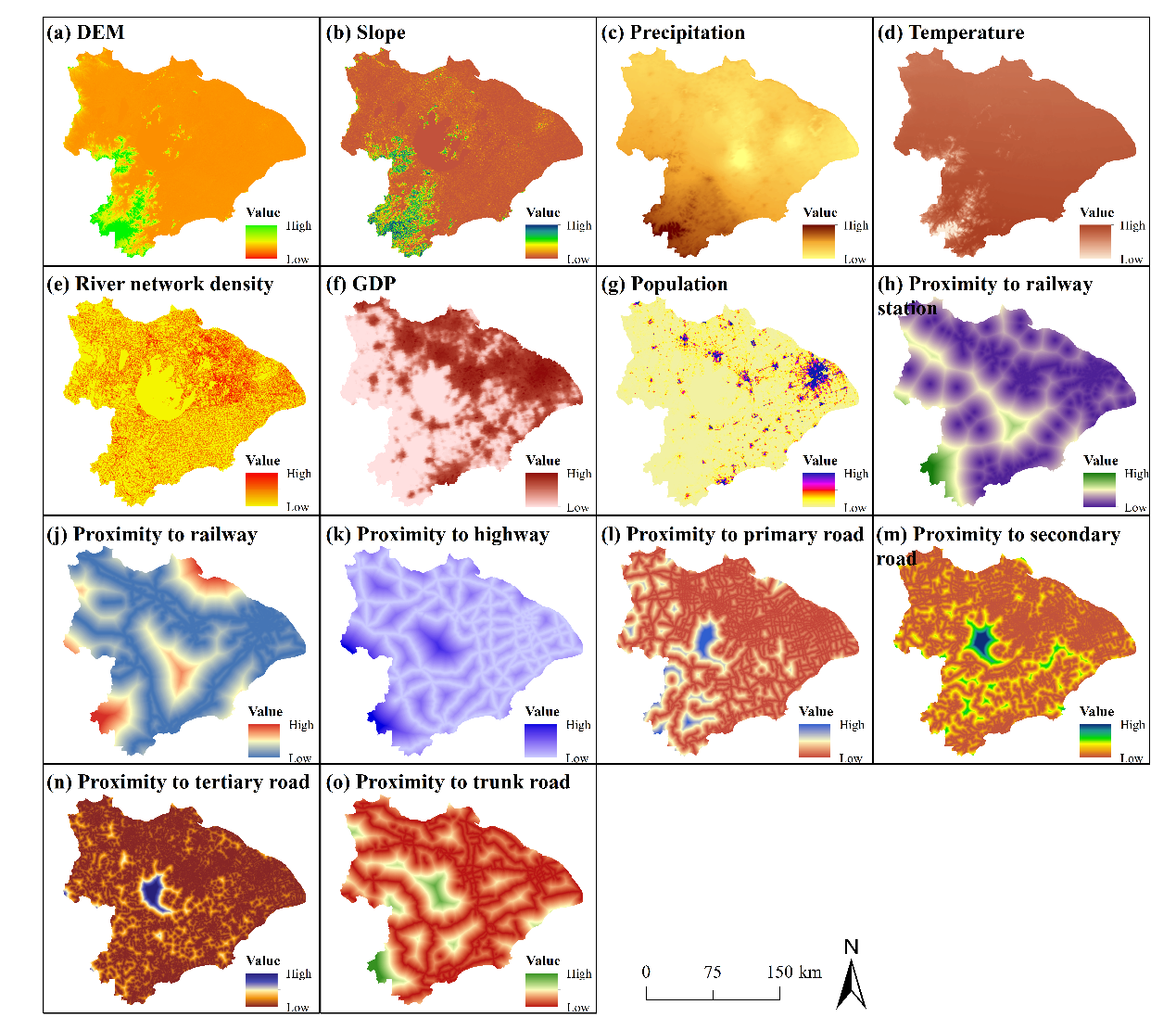


**Fig. S2** Spatial distribution of driving factors affecting land use.


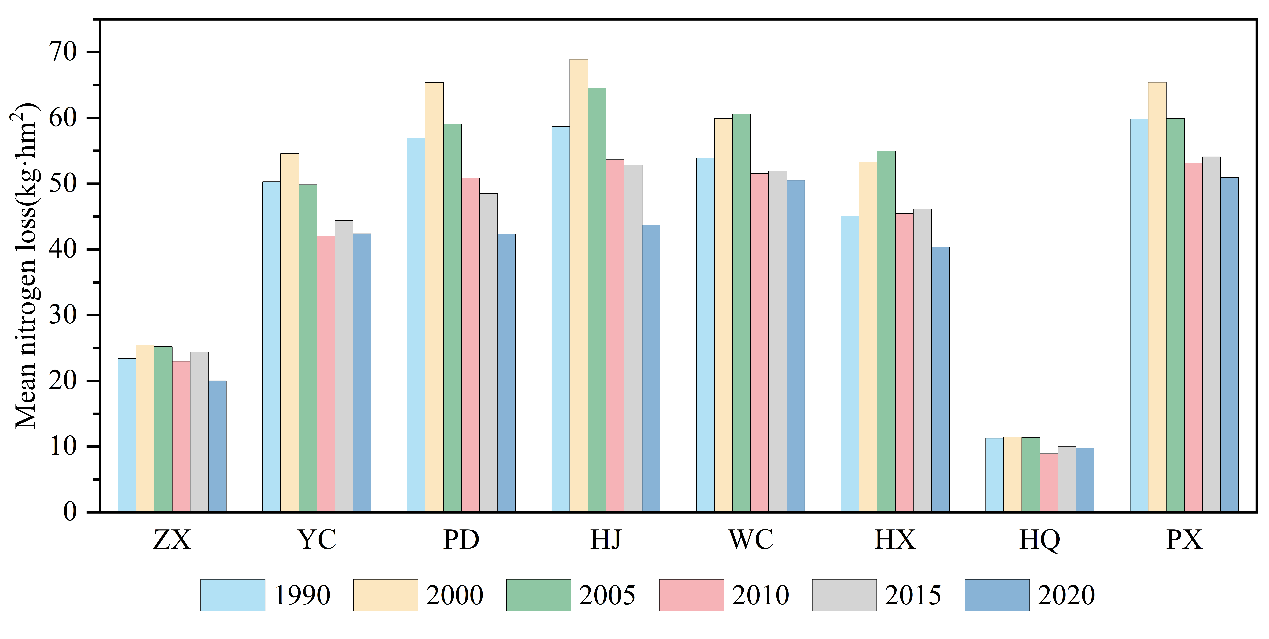


**Fig. S3** Mean nitrogen loss from sub-basins; WC, HX, ZX, HQ, YC, PX, PD, HJ represent eight sub-basins.

**
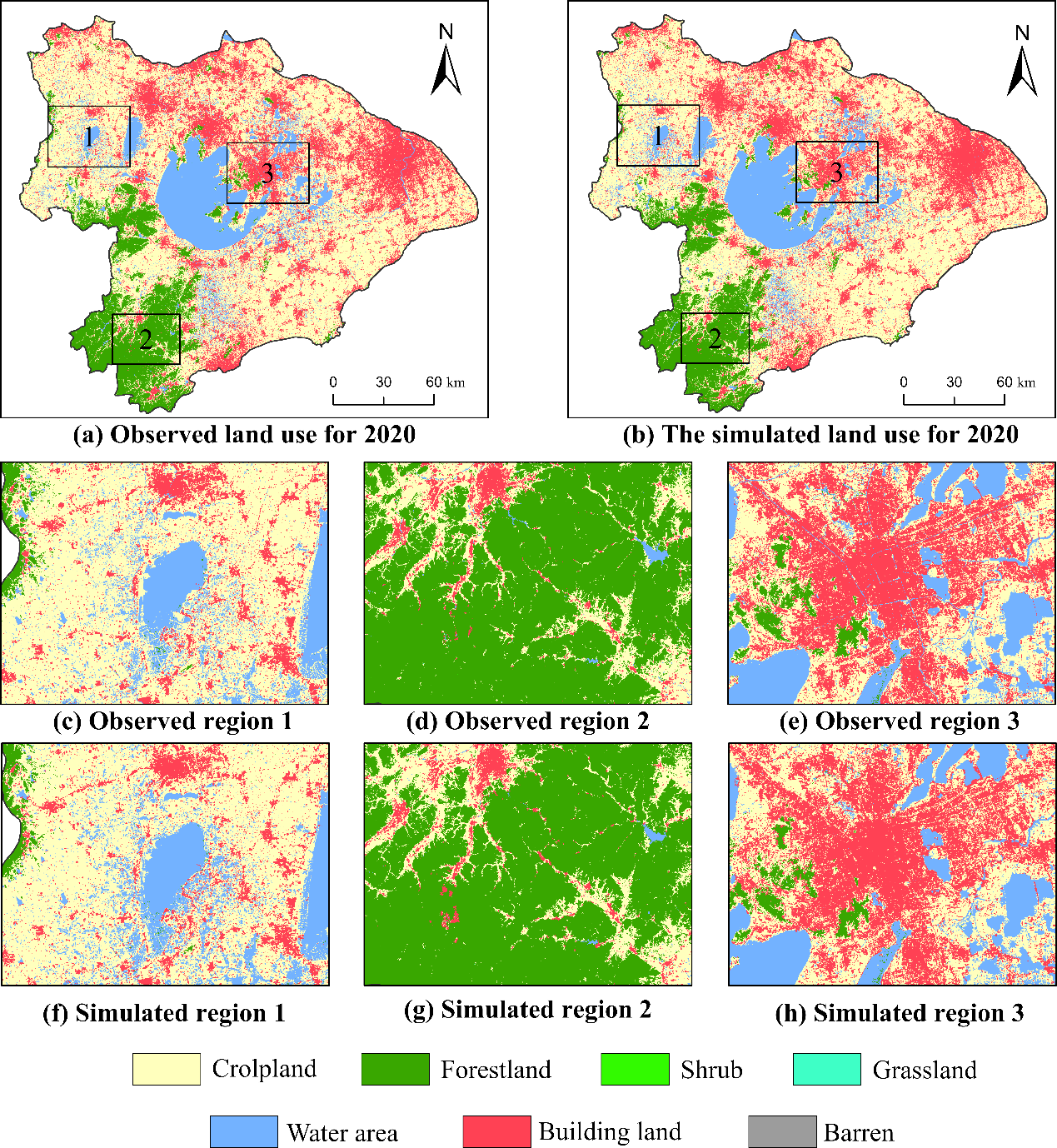
**

**Fig. S4** Comparison of observed land use and simulated land use.


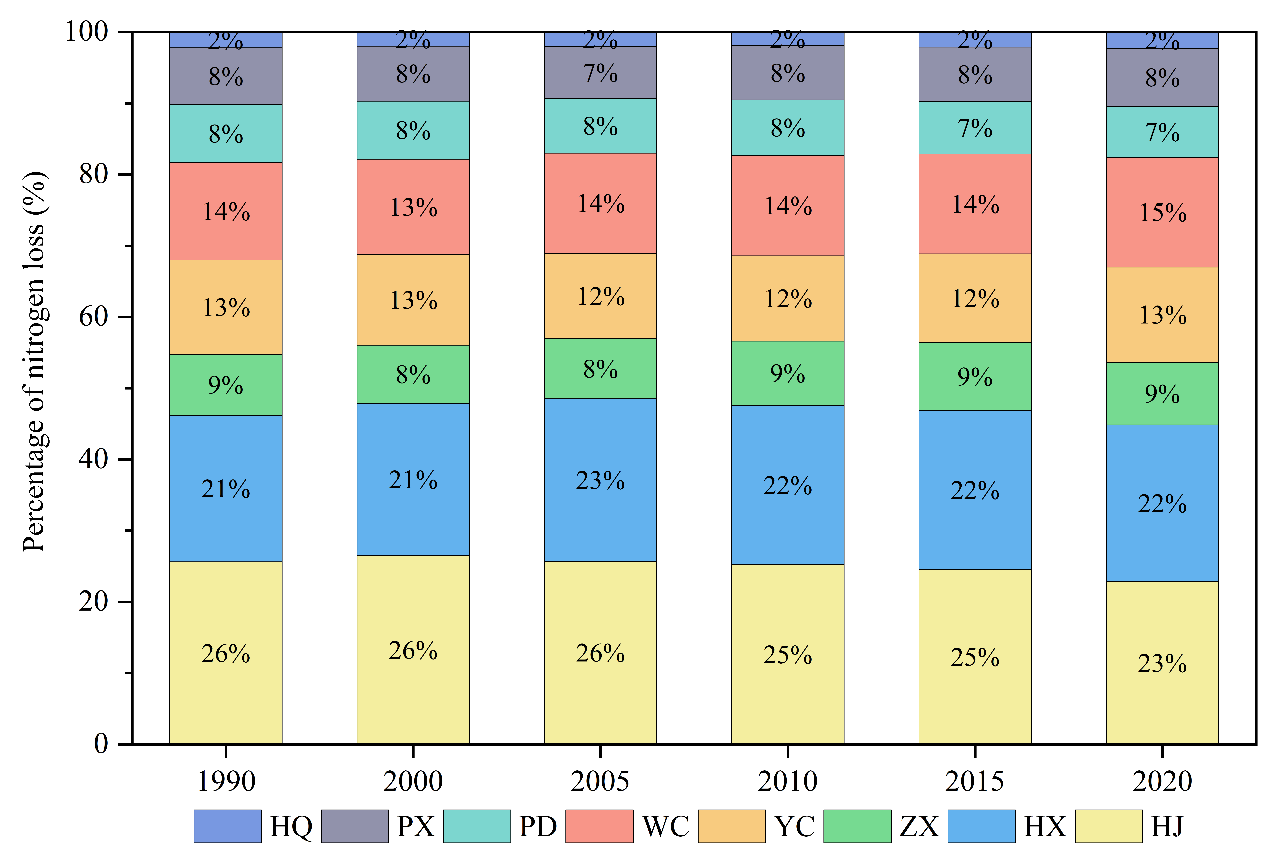


**Fig. S5** Percentage of nitrogen loss from sub-basins; WC, HX, ZX, HQ, YC, PX, PD, HJ represent eight sub-basins.


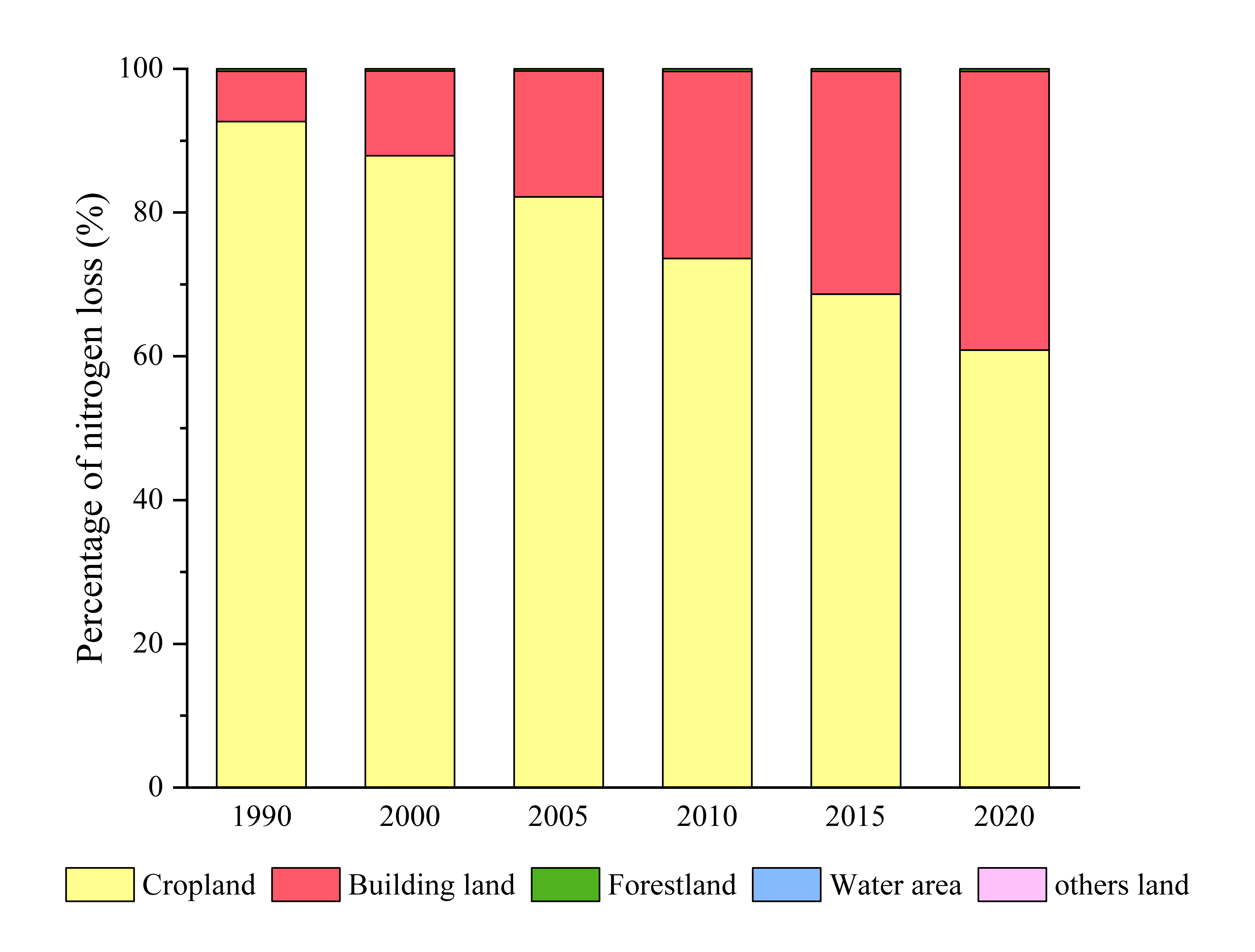
**Fig. S6** Percentage of nitrogen loss from different land use types.

**References**

Liang, X., Guan, Q., Clarke, K. C., Liu, S., Wang, B., Yao, Y., 2021. Understanding the drivers of sustainable land expansion using a patch-generating land use simulation (PLUS) model: A case study in Wuhan. China. Comput., Environ. UrbanSyst. 85, 101569. <https://doi.org/10.1016/j.compenvurbsys.2020.101569>

Ministry of Ecology and Environment of the People’s Republic of China, 20002. Environmental quality standards for surface water (GB3838-2002). <https://www.mee.gov.cn/ywgz/fgbz/bz/bzwb/shjbh/shjzlbz/200206/t20020601_66497.shtml>. (accessed 10 November 2022)

Ministry of Agriculture and Rural Affairs, PRC, 2022. The recent Action Plan for Fertilizer Reduction by 2025. <http://www.moa.gov.cn/govpublic/ZZYGLS/202212/t20221201_6416398.htm> (accessed 10 November 2022).

National Development and Reform Commission, PRC, 2008. Overall program on integrated regulation of Taihu Lake Basin. <https://www.ndrc.gov.cn/fggz/hjyzy/hjybh/200806/P020190911468753017581.pdf>. (accessed 10 November 2022).

National Development and Reform Commission, PRC, 2013. Overall program on integrated regulation of Taihu Lake Basin. <https://www.ndrc.gov.cn/fzggw/jgsj/dqs/sjdt/201401/t20140114_1056776.html?code=&state=123> (accessed 10 November 2022).

National Development and Reform Commission, PRC, 2016. Yangtze River Delta Urban Agglomeration Development Plan. <https://www.ndrc.gov.cn/xxgk/zcfb/ghwb/201606/t20160603_962187_ext.html> (accessed 10 November 2022).

Petersen, R.J., Blicher-Mathiesen, G., Rolighed, J., Estrup, H., Andersen, Kronvang, B., 2021. Three decades of regulation of agricultural nitrogen losses: Experiences from the Danish Agricultural Monitoring Program. Sci. Total. Environ. 787, 147619. <https://doi.org/10.1016/j.scitotenv.2021.147619>

R Core Team, 2020. R: a language and environment for statistical computing. R Foundation for Statistical Computing, Vienna, Austria. <https://www.R-project.org/>

Sharp, R., Douglass, J., Wolny, S., Arkema, K., Bernhardt, J., Bierbower, W., Chaumont, N., Denu, D., Fisher, D., Glowinski, K., Griffin, R., Guannel, G., Guerry, A., Johnson, J., Hamel, P., Kennedy, C., Kim, C.K., Lacayo, M., Lonsdorf, E., Mandle, L., Rogers, L., Silver, J., Toft, J., Verutes, G., Vogl, A.L., Wood, S., Wyatt, K., 2020. InVEST 3.12.0.post3+ug. gd99d637 User's Guide. The Natural Capital Project, Stanford University, University of Minnesota, The Nature Conservancy, and World Wildlife Fund

TBAMWR, 2019. Report on the Health of Taihu Lake. <http://www.tba.gov.cn/slbthlyglj/thjkzkbg/content/slth1_09f7d6b21629439f9891c7fd70ad49d8.html> (accessed 10 November 2022)

TBAMWR, 2021. Water Resources Bulletin of Taihu Lake Basin. <http://www.tba.gov.cn/slbthlyglj/szygb/content/c2844cb0-c8b9-48e9-b382-ea4a2f3ba291.html> (accessed 10 November 2022)

The United Nations World Water Development Report, 2018. World Water Assessment Programme (Nations Unies). <https://www.unwater.org/publications/world-water-development-report-2018/.%20(2018)> (accessed 1 December 2022)

Wang, Y., Yang, G., Li, B., Wang, C., Su, W.Z., 2022. Measuring the zonal responses of nitrogen output to landscape pattern in a flatland with river network: a case study in Taihu Lake Basin, China. Environ Sci Pollut Res. 29, 34624–34636. https://doi.org/10.1007/s11356-021-15842-x

Xia, Y., Ti, C., She, D., Yan, X., 2016. Linking river nutrient concentrations to land use and rainfall in a paddy agriculture-urban area gradient watershed in southeast China. Sci. Total. Environ. 566, 1094–1105. https://doi.org/10.1016/j.scitotenv.2016.05.134

Xiong, C.H., Wang, G.L., Xu, L.T., 2021. Spatial differentiation identification of influencing factors of agricultural carbon productivity at city level in Taihu lake basin, China. Sci. Total. Environ. 80, 149610. https://doi.org/10.1016/j.scitotenv.2021.149610

Yang, J., Huang, X., 2021. The 30 m annual land cover dataset and its dynamics in China from 1990 to 2019, Earth Syst. Sci. Data. 13, 3907–3925. <https://doi.org/10.5194/essd-13-3907-2021>.

1. * Corresponding author at: School of Geography, Nanjing Normal University, 1 Wenyuan Road, Qixia, Nanjing 210023, China.

   *E-mail address*: wangyanhua@njnu.edu.cn (Y.H. Wang). [↑](#footnote-ref-1)
